# Somatic mutation detection and classification through probabilistic integration of clonal population information

**DOI:** 10.1038/s42003-019-0291-z

**Published:** 2019-01-31

**Authors:** Fatemeh Dorri, Sean Jewell, Alexandre Bouchard-Côté, Sohrab P. Shah

**Affiliations:** 1 Department of Computer Science, University of British Columbia, 201- 2366 Main Mall, V6T 1Z4 Vancouver, Canada; 2 Department of Statistics, University of Washington, B313 Padelford Hall, Northeast Stevens Way, Seattle, WA 24105 USA; 3 Department of Statistics, University of British Columbia, 3182 Earth Sciences Building, 2207 Main Mall, V6T 1Z4 Vancouver, Canada; 4 Department of Molecular Oncology, University of British Columbia, 675 West 10th Avenue, V5Z 1L3 Vancouver, Canada; 5 Department of Pathology and Laboratory Medicine, University of British Columbia, Rm. G227 - 2211 Wesbrook Mall, 24105 Vancouver, Canada; 6 Computational Oncology, Department of Epidemiology and Biostatistics, Memorial Sloan Kettering Cancer Center, Kettering Cancer Center, 417 E 68th Street, New York, NY 10065 USA

## Abstract

Somatic mutations are a primary contributor to malignancy in human cells. Accurate detection of mutations is needed to define the clonal composition of tumours whereby clones may have distinct phenotypic properties. Although analysis of mutations over multiple tumour samples from the same patient has the potential to enhance identification of clones, few analytic methods exploit the correlation structure across samples. We posited that incorporating clonal information into joint analysis over multiple samples would improve mutation detection, particularly those with low prevalence. In this paper, we develop a new procedure called MuClone, for detection of mutations across multiple tumour samples of a patient from whole genome or exome sequencing data. In addition to mutation detection, MuClone classifies mutations into biologically meaningful groups and allows us to study clonal dynamics. We show that, on lung and ovarian cancer datasets, MuClone improves somatic mutation detection sensitivity over competing approaches without compromising specificity.

## Abstract (web summary)

Fatemeh Dorri et al. present MuClone, a method for simultaneous detection and classification of mutations across multiple tumor samples derived from one patient. They show that clonal information improves sensitivity in detecting somatic mutations.

## Introduction

Genomic accumulation of somatic point mutations, or single nucleotide variants (SNVs), can disrupt the regular activity of cells and result in cancer initiation and progression. Collectively, the complete repertoire of SNVs across a cancer genome (numbering in the thousands) form a statistically robust marker for inferring clonal populations and studying tumour evolution. As such, accurate detection of all somatic SNVs, including those with low prevalence, is vital as they can define clones with phenotypic properties of interest. Mechanistic association of specific clones with properties such as treatment resistance, metastatic potential, and fitness under therapeutic selective pressures remains a key objective of biomedical investigators studying tumour progression.

Phylogenetic analysis can encode the evolutionary lineage of tumour cells across time and anatomic space^1–7^. Sequencing of multiple samples of a cancer to reconstruct evolutionary patterns and drug response profiles are increasingly common. For example, in rapid autopsy programs, at the time of a patient’s death, tens to hundreds of metastatic samples are collected for future study^8,9^. Recent multi-sample sequencing studies in renal, lung, ovary, breast, colorectal, and other cancers have revealed striking evolutionary and clinically important properties of cancers^5,7,10,11^. However, the analytical methods to detect mutations from such experimental designs are still immature, and few studies have leveraged shared statistical strength across samples to detect mutations with greater sensitivity.

In the limit case, all cells likely harbour unique genomes, however due to the nature of branched evolutionary processes, clones can be coarsely modelled as major clades in the cell lineage phylogeny of a cancer. These clades share the majority of mutations, and therefore define first approximations to the genotypes of clones. Clonal genotypes and their relative abundances in the cancer cell population can be approximated by clustering mutations measured in bulk tissues and estimating their cellular prevalences (the variant tumour cell fraction)^12,13^.

Phylogenetic algorithms mostly use mutations (represented as binary genetic markers), as inputs to infer the branched evolutionary lineages of tumour cells^14,15^. Thus, mutation detection accuracy will ultimately impact the performance of phylogenetic inference algorithms.

Detection of low prevalence mutations is a major challenge due to typically small signal to noise ratios, owing to: (i) contamination by normal cells; (ii) genome copy number alteration; and (iii) the presence of mutations in only a small fraction of tumour cells (intra-tumour heterogeneity). In this work, we illustrate that knowledge of the clonal population structure improves detection of mutations defining low prevalence clonal genotypes.

Although SNV calling algorithms are ubiquitous in the literature, it remains challenging to detect low prevalence mutations. Algorithms have been developed for calling mutations from a single sample^16,17^, paired (matched normal and tumour) samples^18–22^, or multiple samples^23,24^. We list several popular algorithms. Mutationseq uses a feature based classifier for calling mutations^20^, where the features are constructed from matched paired normal and tumour samples. Strelka is a method for somatic SNV and small indel detection from sequencing data of matched normal and tumour samples^18^. It is based on a Bayesian approach that uses normal and tumour samples’ allele frequencies with normal expected genotype structure. MuTect uses a Bayesian classifier that employs various filters to ensure high specificity to detect mutations from matched normal and tumour samples^21^. FreeBayes uses short read alignments to call the most likely genotypes for the population at each position. It can be run in single mode using only one tumour sample or in multiple mode utilizing multiple tumour samples from the same patient^25^. FreeBayes can detect somatic mutations if germlines are manually removed. MultiSNV jointly analyses all available samples under a Bayesian framework to improve the performance of calling shared mutations^23^. SNV calls from GATK^26^ are refined and corrected by using phylogeny information across multiple samples^24,27^.

In our proposal, MuClone, we exploit prior knowledge of tumour content, tumour cellular prevalence, and copy number information across multiple samples to improve detection of somatic SNVs, and in particular, low prevalence ones. Our model uses mutation clusters and copy number information obtained from standard approaches^28,29^. In the first step, a set of stringent SNV calls or validated SNVs (using targeted sequencing data) is used to infer mutation cluster information. Then, MuClone uses the inferred cluster information to more accurately call mutations across genome (whole genome or exome sequencing data). In addition to calling mutations, MuClone also classifies mutations into clusters based on cellular prevalence. This provides the user with the opportunity to profile mutation changes across time and space, and adds a rich layer of interpretation into the detection process.

We test MuClone through simulation studies and an application to real, multiple sample, patient data. These experiments reveal that incorporating the cellular prevalences of different clusters improves accuracy. Moreover, in real data, MuClone exhibits higher sensitivity (true positive rate or recall) in detecting mutations without compromising specificity (true negative rate) compared with other methods.

## Results

### Synthetic data

In this section, we examine the performance of MuClone on simulated data. In what follows, we generate *N* loci from *M* samples with *K* underlying tumour mutation clusters with sequencing error rate $\epsilon$ and tumour content *t*_*m*_.

We first randomly generate an evolutionary relationship between clusters, viewed as a binary phylogenetic tree. Each node in the tree represents a mutation cluster. The root node represents the ancestral cluster. For each sample, the cellular prevalence of the first descendant, ${\phi} _{1^{\mathrm{st}}}$, is sampled from a Uniform distribution over [0, *ϕ*_parent_], where *ϕ*_parent_ is the cellular prevalence of the parent node (cluster). The cellular prevalence of the second descendant, ${\phi} _{2^{\mathrm{nd}}}$, is sampled from a Uniform distribution over $[ {0,{\phi} _{{\mathrm{parent}}} - {\phi} _{1^{\mathrm{st}}}}]$, defined so that the sum of the children’s prevalences do not exceed their parent’s cellular prevalence. The absence or presence of each cluster, in each sample, is sampled from a Bernoulli distribution that assigns equal probability to both outcomes. If a cluster is not present in a sample, the corresponding cellular prevalence will be 0. See Supplementary Fig. 1 for an example of this process. Loci are assigned to a cluster uniformly at random from {0, …, *K*}, where cluster 0 represents the wildtype cluster and {1, …, *K*} are mutation clusters. For each locus, in each sample, the number of reads overlapping the locus (depth) is sampled from a Poisson distribution with mean *d*_*m*_. Wildtype copy number is deterministically set to 2, and a copy number profile (major and minor copy number) is generated according to the following steps: The total copy number, *C*^*t*^, is sampled uniformly at random from {1, …, *C*_max_}. An integer number, *C*^*b*^, is randomly (following a discrete Uniform distribution) picked from 1 to *C*^*t*^, and *C*^*a*^ is defined as *C*^*a*^ = *C*^*t*^ − *C*^*b*^. Lastly, the major copy number is set to the maximum of *C*^*b*^ and *C*^*a*^; the minor copy number is set to the minimum of those two values. Then, corresponding to each cluster, the number of variant reads are sampled from the Beta-Binomial distribution described in Eq. (7) with precision parameter equal to 1000.

### Synthetic data evaluation

We simulated 10 synthetic data for 20,000 loci from 4 samples of a patient, with 5 underlying clusters, including an ancestral cluster. The maximum copy number was 5, and the error rate was 0.01. The average sequencing depth was assumed to be 100 for all samples.

**Fig. 1:**
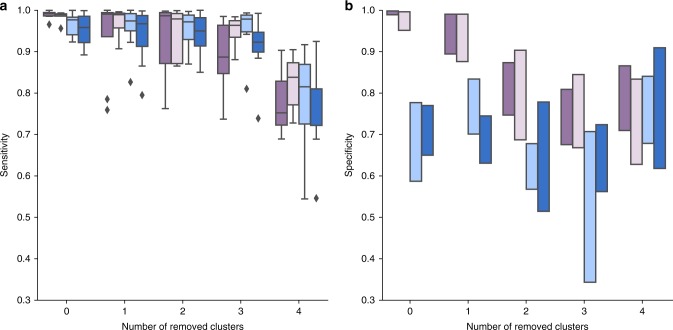
MuClone's performance with inaccurate clonal information: 10 synthetic datasets generated for 20,000 loci, from 4 samples of a hypothetical patient, with 5 underlying clusters. The maximum copy number is 5, error rate is set to 0.01, and average sequencing depth is approximately 100. To assess performance, we add noise from a mean zero normal with standard deviation equal to 0 (dark purple), 0.01 (light purple), 0.1 (light blue), and 0.2 (dark blue) to the cellular prevalence and also remove the clonal information of different number of clusters. **a** Sensitivity and **b** Specificity of MuClone with parameters: wildtype prior = 0.5, *Φ*_*T*_ = 0.02, error rate = 0.01, tumour content = 0.75, and precision parameter = 1000

To assess the performance and robustness of MuClone, we systematically shielded MuClone from clonal information (Fig. 1). In particular, the cellular prevalence information was perturbed by (i) adding noise to its value, or (ii) removing the cellular prevalence information of the clusters. The noise was generated from a normal distribution with mean zero and standard deviations: 0, 0.01, 0.1, and 0.2. The noise value, *ν*, was added to the cellular prevalence of the cluster, while bounding the resulting value between 0 and 1, that is,

$${\phi} _m^{ \ast z} = {\mathrm{min}}\left( {{\mathrm{max}}({\phi} _m^z + \nu ,0),1} \right),$$

where ${\phi} _m^{ \ast z}$ and ${\phi} _m^z$ are the perturbed and original cellular prevalence of cluster *z* and sample *m*, respectively. The clusters which their clonal information was removed, were randomly chosen with equal probabilities. As expected, both sensitivity and specificity were highest with complete and accurate clonal information; see Fig. 1. This suggests that incorporating clonal information can improve mutation detection accuracy and gives evidence to support MuClone’s approach. Furthermore, since the sensitivities were only marginally impacted by adding noise to the clonal information, MuClone should be able to cope with modest misspecification of the prior. However, specificity can decrease if the cellular prevalence is reduced to levels associated with the wildtype cluster and sensitivity can improve if adding noise increases the cellular prevalence to levels associated with a removed mutation cluster.

Naturally, accuracy is most severely impacted with reduced/corrupted clonal information; see Fig. 1. For the modest level of noise (noise standard deviation 0 and 0.01), the sensitivity and specificity of removing various numbers of clusters were compared through a Kruskal–Wallis test (4e−5 ≤ *p*-values ≤ 1e−4) which shows that the change in performance due to clonal information is significant. In noiseless settings, the confidence interval for the difference (of zero and four removed clusters) in mean sensitivity and specificity are [0.16, 0.26] and [0.08, 0.32], respectively. When the noise standard deviation is equal to 0.01, these intervals are [0.11, 0.21] and [0.09, 0.36].

**Fig. 2:**
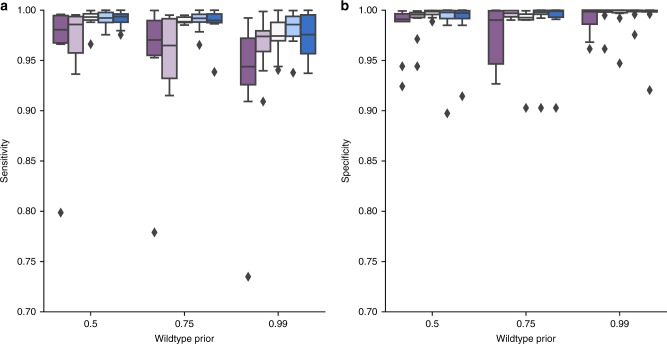
MuClone’s performance as a function of wildtype prior: 10 synthetic datasets generated for 20,000 loci, from 4 samples of a hypothetical patient, with 5 underlying clusters. The maximum copy number is 5 and the error rate is set to 0.01, and average sequencing depth is approximately 100. We assess the performance for *Φ*_*T*_ equal to 0.001 (dark purple), 0.01 (light purple), 0.02 (white smoke), 0.03 (light blue), 0.04 (dark blue). **a** Sensitivity and **b** Specificity of MuClone with parameters: error rate = 0.01, tumour content = 0.75, and precision parameter = 1000

We also explored how sensitivity and specificity change as a function of the wildtype prior and the threshold *Φ*_*T*_ used to distinguish the cellular prevalence cutoff of a mutation cluster. In Fig. 2, we tested MuClone with wildtype prior values 0.5, 0.75, and 0.99, and with *Φ*_*T*_ values 0.001, 0.01, 0.02, 0.03, and 0.04. In the case that the wildtype prior equals 0.5, we assumed that a locus is equally likely to be a mutation or not (when no other information is provided). MuClone’s sensitivity and specificity are near 1 for *Φ*_*T*_ equal to 0.02 and wildtype prior equal to 0.5. As expected, with small values of *Φ*_*T*_, the sensitivity and specificity decrease since it is difficult to distinguish between wildtypes and mutations with small cellular prevalences. The sensitivity also decreases for large values of *Φ*_*T*_ because mutations are miscalled as wildtypes. When the error rate was 0.01, and wildtype prior was 0.5, the optimal *Φ*_*T*_ was about 0.02. We used these values for the following experiments.

**Fig. 3:**
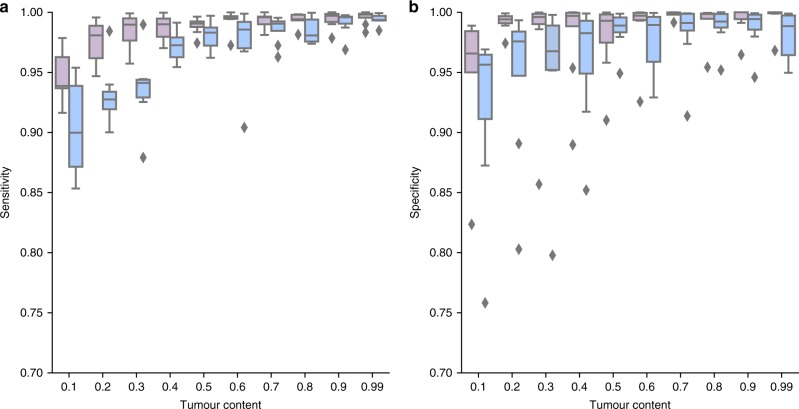
MuClone’s performance as a function of tumour content: 10 synthetic datasets generated for 20,000 loci, from 4 samples of a hypothetical patient, with 5 underlying clusters. The maximum copy number is 5, and average sequencing depth is approximately 100. We assess the performance for error rate equal to 0.001 (light purple) and 0.01 (light blue), and tumour content from 0.1 to 0.99. **a** Sensitivity and **b** Specificity of MuClone with parameters: wildtype prior = 0.5, *Φ*_*T*_ = 0.02, and precision parameter = 1000

The performance of MuClone was tested with various tumour content (from 0.1 to 0.99) and different error rates (0.01 and 0.001); see Fig. 3. For samples with tumour content greater than 0.5, the sensitivity and specificity remain close to 1. Sensitivity and specificity decreased to only about 0.9 when the tumour content in the sample is as low as 0.1. These results establish promising performance over different ranges of tumour content with different error rates (likely scenarios in real data).

**Fig. 4:**
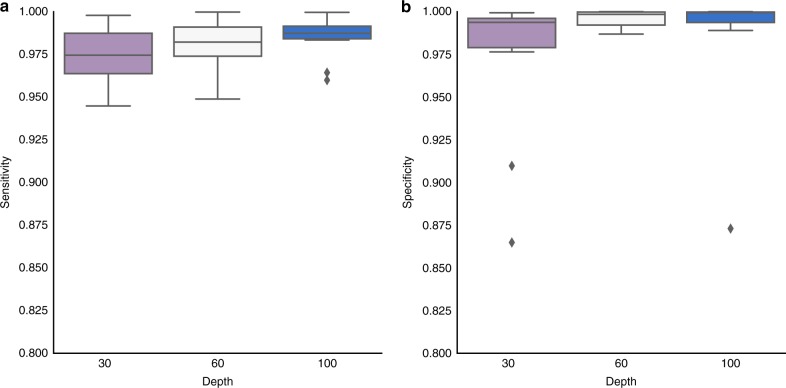
MuClone’s performance as a function of different depth: 10 synthetic datasets generated for 20,000 loci, from 4 samples of a hypothetical patient, with 5 underlying clusters. The maximum copy number is 5, error rate is set to 0.01 and average sequencing depth is approximately 100. **a** Sensitivity and **b** Specificity of MuClone with parameters: wildtype prior = 0.5, *Φ*_*T*_ = 0.02, error rate = 0.01, tumour content = 0.75, and precision parameter = 1000

In addition, we also explored the performance of MuClone for samples with different coverage (mean depth): 30, 60 and 100; see Fig. 4. Intuitively, the performance is higher when we have more coverage. Since MuClone leverages cellular prevalence information to improve the performance of mutation detection, the performance gain is noticeable when the variant allelic ratio resolution supports the given cellular prevalence resolution (in our analysis the cellular prevalence of mutations is greater than 0.02).

**Fig. 5:**
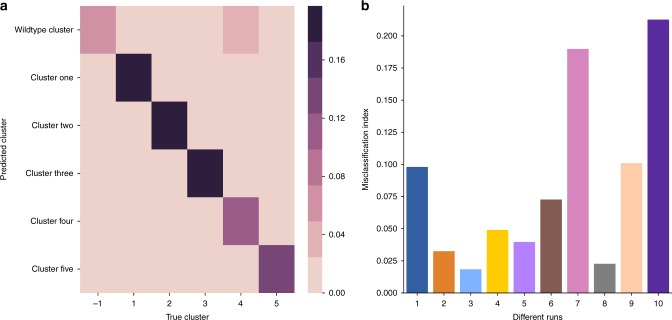
MuClone’s classification performance: 10 synthetic datasets generated for 20,000 loci, from 4 samples of a hypothetical patient, with 5 underlying clusters. The maximum copy number is 5, error rate is set to 0.01. **a** Bin (*i*, *j*) shows the fraction of mutations in cluster *i* that are classified into cluster *j* by MuClone. 85% of the mutations are classified correctly. **b** Misclassification index for 10 independent samples. MuClone with parameters: wildtype prior = 0.5, *Φ*_*T*_ = 0.02, error rate = 0.01, tumour content = 0.75, and precision parameter = 1000

Figure 5 demonstrates how well mutations are classified by MuClone. The input clonal information was perturbed by adding noise from zero mean normal distribution with standard deviation 0.01 to simulate a more realistic scenario. In Fig. 5a, each bin (*i*, *j*) shows the fraction of mutations in cluster *i* that are classified into cluster *j* by MuClone. Figure 5a shows that 85% of mutations are classified into the correct cluster.

In order to show that the classification errors occurred between clusters with small phylogenetic distance, we define a misclassification index to quantify phylogenetic distance; calculated as

1 $${\mathrm{Misclassification}}\,{\mathrm{index}} = \frac{{\mathop {\sum}\nolimits_{i \ne j} {\kern 1pt} q_{(i,j)} \times \frac{{{\mathrm{dist}}_{(i,j)} - {\mathrm{dist}}_i^{{\mathrm{min}}}}}{{{\mathrm{dist}}_i^{{\mathrm{max}}} - {\mathrm{dist}}_i^{{\mathrm{min}}}}}}}{{\mathop {\sum}\nolimits_{i \ne j} {\kern 1pt} q_{(i,j)}}},$$

where *q*_(*i*,*j*)_ is the number of mutations in cluster *i* that are classified into cluster *j*, and the Euclidean distance between the cellular prevalences of cluster *i* and *j* is denoted by dist_(*i*,*j*)_. The distance of the closest and farthest cluster to cluster *i* is denoted by $\mathrm{dist}_i^{{\mathrm{min}}}$ and $\mathrm{dist}_i^{{\mathrm{max}}}$, respectively. In Fig. 5b, small misclassification indices demonstrate that misclassified mutations occur between close clusters. This can be interpreted as phylogenetically recently separated clusters.

### Real data

Two real data sets with multiple samples for each patient were used to evaluate the performance of MuClone. The first data set was multiple whole genome sequencing data from 7 patients with high grade serous ovarian cancer. The second data set was multiple whole exome sequencing data from 8 patients with non-small-cell lung cancer (NSCLC).

### High grade serous ovarian cancer

We tested MuClone’s performance on whole genome sequencing data (with depth 30x) from multiple tumour samples surgically resected from high grade serous ovarian cancer patients^5^. The samples were obtained from different spatially distributed metastatic sites. Brief details about the number of samples for each patient, sample sites and the number of validated loci for each patient are shown in Supplementary Table 1. Germline mutations were excluded from the list.

The copy number, tumour purity, and mutation cluster information for experimentally re-validated mutation status were taken from the phylogenetic study of high-grade serous ovarian cancer (see the supplementary note of the paper^5^). Mutation clusters were estimated with PyClone^12^ on the deep targeted sequencing data (>1000x coverage) from the same samples and in three patients with accompanying single cell sequencing data (see Table S16 in the phylogenetic study of high-grade serous ovarian cancer paper^5^). Copy number and tumour purity estimates were calculated with the TITAN software^28^. In order to eliminate germlines, loci with variant nucleotides in the corresponding normal sample were removed from the dataset. Then, the performance of MuClone was benchmarked against Strelka^18^(v2.0.15), MutationSeq^20^(v4.3.7), MuTect^30^, FreeBayes^25^(v1.2.0-2), MultiSNV^23^ and naive MuClone. Naive MuClone is a version of MuClone where no clonal information is provided (that is, all mutations are from an ancestral cluster).

**Fig. 6:**
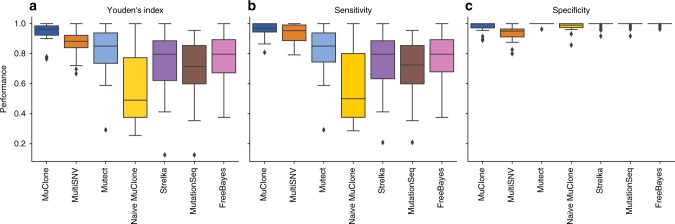
Performance comparison of different methods on whole genome sequencing data from patients with high grade serous ovarian cancer. **a** Youden’s index, **b** Sensitivity, and **c** Specificity across different mutation detection methods (from left to right: MuClone (dark blue), MultiSNV (orange), MuTect (light blue), Naive MuClone (yellow), Strelka (purple), MutationSeq (brown), and FreeBayes (pink)). MuClone parameters are: wildtype prior = 0.5, *Φ*_*T*_ = 0.02, tumour content = 0.75, error rate = 0.01, and precision parameter = 1000

In Fig. 6, the performance of MuClone is compared with other methods executed with default settings. For each patient, *p*, we assessed performance by averaging Youden’s index, sensitivity, and specificity across different samples. For patient *p*, with *n*_*p*_ samples, these are calculated as

$${\mathrm{Sensitivity}}_p = \frac{1}{{n_p}}\mathop {\sum}\limits_{i = 1}^{n_p} {\kern 1pt} {\mathrm{Sensitivity}}_p^i,$$

2 $${\mathrm{Specificity}}_p = \frac{1}{{n_p}}\mathop {\sum}\limits_{i = 1}^{n_p} {\kern 1pt} {\mathrm{Specificity}}_p^i,$$

$${\hbox{Youden's index}}_p = \frac{1}{{n_p}}\mathop {\sum}\limits_{i = 1}^{n_p} \left( {{\mathrm{Sensitivity}}_p^i + {\mathrm{Specificity}}_p^i - 1} \right),$$

where ${\mathrm{Sensitivity}}_p^i$, ${\mathrm{Specificity}}_p^i$ and ${\hbox{Youden's index}}_{p}^{i}$ are the sensitivity, specificity and Youden’s index of sample *i* and patient *p*, respectively. In aggregate, MuClone outperforms other methods by improving sensitivity without compromising specificity; see Fig. 6. False negatives arise mainly because the WGS data is under-represented (the average depth of the WGS data is about 30x) and lacks variant alleles that are present in the targeted sequencing data. False positives arise due to erroneous signal from sequencing technical artefacts.

In Fig. 6, Strelka, MutationSeq, MuTect and Naive MuClone have lower performance as they do not incorporate information across multiple samples. FreeBayes was run on multiple samples and germlines were removed manually, but since the method only considers tumour samples, it has the lower performance versus other methods.

To assess the performance of MuClone and MultiSNV, we conducted a two-sided *t*-test for the difference in the mean of Youden’s index evaluated on mutation calls from MuClone and MultiSNV. The 95% confidence interval is [0.03, 0.1], with *p*-value equal to 0.0006; this shows that the difference is statistically significant. Importantly, MuClone improves sensitivity, enabling the detection of more mutations across the whole genome.

**Fig. 7:**
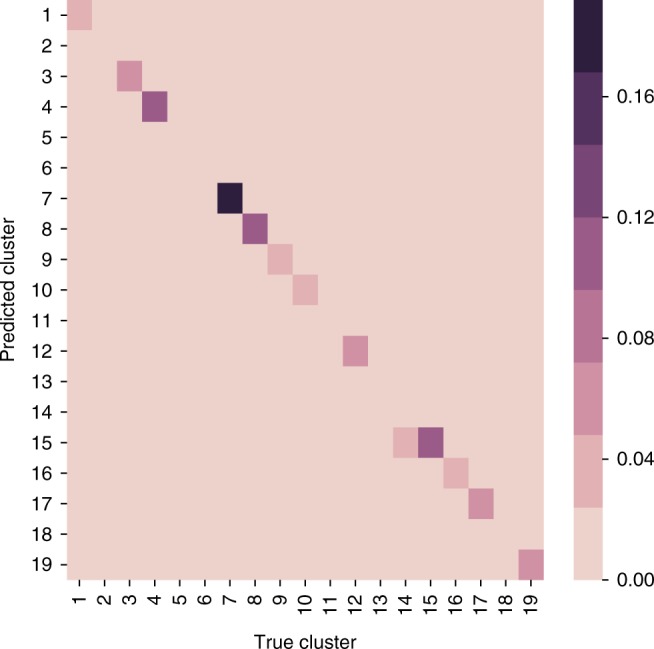
Classification of 153 mutations of patient 1 with high grade serous ovarian cancer across 6 samples. Bin (*i*, *j*) shows the fraction of mutations in cluster *i* that are classified into cluster *j* by MuClone. MuClone parameters are: wildtype prior = 0.5, *Φ*_*T*_ = 0.02, error rate = 0.01, tumour content = 0.75, and precision parameter = 1000. 93% of the elements are diagonal

Figure 7 depicts the classification of mutations into clusters relative to the ground truth, as defined by running PyClone on the data (omitting singleton clusters^5^). Each bin (*i*, *j*) of Fig. 7 shows the fraction of mutations in cluster *i* that are classified into cluster *j* by MuClone; 93% are correctly classified by MuClone. Moreover, we notice that misclassified mutations are classified into phylogenetically similar clusters (the misclassification index for patient 1 was 0.015).

### Non-small-cell lung cancer

We tested MuClone’s performance on early-stage NSCLC samples from the TRACERx data set^7^. To help obtain the clonal and subclonal census, multiple tumour regions for each patient were sequenced by Illumina HiSeq. We used the copy number, purity estimate, and the mutation cluster information available in TRACERx study Supplementary Material^7^. In the TRACERX study, the cellular prevalence was calculated from the whole exome sequencing data on a set of stringent mutations that were selected from MuTect and VarScan2 results with post-processing. In addition, the TRACERx study added a few mutations to reduce missed subclonal mutations; see Supplementary Appendix of TRACERx study^7^.

To compare the performance of MuClone with Strelka, MultiSNV, and MuTect, we randomly selected 8 patients with subclonal mutations from the TRACERx data set (see Table S2 Supplementary Appendix 1 of the paper^7^). The TRACERx study generated a re-validated and curated list of mutations for their analysis; see Supplementary Appendix 2 of TRACERx study^7^. The mutations with full copy number information across all 8 patients were used as ground truth to evaluate performance.

We evaluated the false negative rates of mutation calling across several methods; see Table 1. Altogether, out of 7238 mutations, MuClone missed 475 mutations while Strelka, MultiSNV and MuTect missed 7205, 5720, and 1086 mutations respectively. Hence, borrowing statistical strength, as done in MuClone, across samples likely increases sensitivity to real mutations.

**Table 1:** Total number of false negative calls, across multiple samples of non-small cell lung cancer patients, from different mutation detection algorithms

Patient	MuClone	MultiSNV	MuTect	Strelka
CRUK0003	52	350	60	430
CRUK0004	16	188	36	240
CRUK0005	212	1736	236	2040
CRUK0013	26	490	270	540
CRUK0062	30	469	42	609
CRUK0063	75	445	40	510
CRUK0065	60	1902	342	2640
CRUK0094	4	140	60	196

**Fig. 8:**
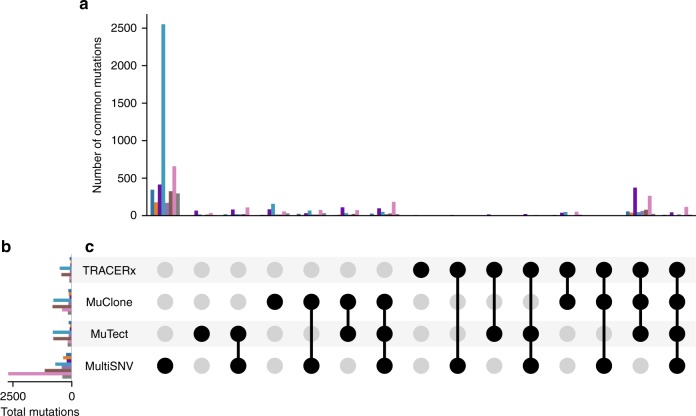
Comparison of detected mutations from MultiSNV, MuTect, MuClone, and TRACERx on whole exome sequencing data from non-small cell lung cancer patients. CRUK0003 (dark blue), CRUK0004 (orange), CRUK0005 (dark purple), CRUK0013 (light blue), CRUK0062 (light purple), CRUK0063 (brown), CRUK0065 (pink), and CRUK0094 (grey). To illustrate overlap in detected mutations from all combinations of different methods, in **a** the number of mutations called in all selected methods, but not in any other method, are plotted for each patient. Dark circles in the columns of **c** indicate selected methods. For example, the first column in **c** indicates mutations that are only called by MultiSNV (all circles are grey except the one corresponding to MultiSNV). **b** displays the total number of mutations called for each method. MuClone removes reads with mapping quality less than 5 and positions which have (i) a variant nucleotide in a normal sample, (ii) more than 40% filtered basecalls; or (iii) more than 75% of the reads that cross the site have deletions in any of the samples. Other methods executed with default settings. See UpSet tool for additional visualization details^31^

We next ran MuClone, MultiSNV and MuTect on the whole exome data from multiple samples of 8 patients to ascertain specificity. We note that MuClone removes reads with mapping quality less than 5 and for positions that have (i) a variant nucleotide in a normal sample, (ii) more than 40% filtered basecalls (A basecall is filtered if more than 3 mismatches occur between the read and the reference within a window of 20 bases on each side of the site.); or (iii) more than 75% of the reads that cross the site have deletions in any of the samples^18^. For exome sequencing data, mutations were called if the corresponding MuClone mutation probability is greater than 0.9. The other methods were executed with default settings. The total number of calls and the number of common calls between different methods (restricted to positions with copy number information) at the patient level is depicted in Fig. 8. A high degree of variation across callers is observed. Altogether, MuClone called 13,556 mutations while MultiSNV and MuTect called 31,374 and 11,915, respectively. MultiSNV output the largest number of calls in all of the samples, while MuTect and MuClone output similar number of calls. Figure 8 also demonstrates the mutations used in the TRACERx study and their overlap with the mutation calls in different methods. The set of mutations overlapping between MuClone and TRACERx is most similar; this suggests that the increase in sensitivity conferred by MuClone does not come at the expense of specificity.

**Fig. 9:**
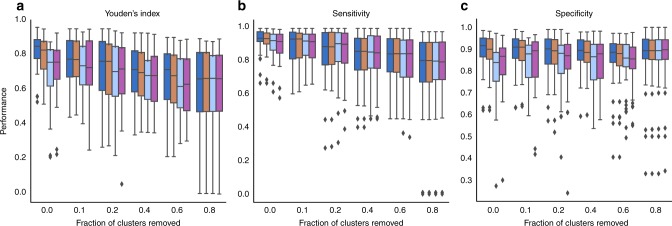
MuClone performance with inaccurate clonal information: The mutations with full copy number information across all 8 non-small cell lung cancer patients are used as ground truth. To asses the performance, we add noise from a zero mean normal with standard deviation equal to 0 (dark blue), 0.01 (orange), 0.1 (light blue), and 0.2 (purple) and also remove the clonal information of different fractions of clusters. **a** Youden’s index, **b** Sensitivity, and **c** Specificity of MuClone with different fraction of clusters information removed

We also explored the performance of MuClone when clonal information differs in the number of input clusters or the value of the cellular prevalence; see Fig. 9. We perturbed the value of the cellular prevalences (estimated by PyClone) by adding noise from a mean zero normal distribution with different standard deviations: 0, 0.01, 0.1, and 0.2. We see that MuClone is robust to slight changes of cellular prevalence values. We also shielded MuClone from different fractions of the clonal information and that decreased the performance more than adding noise. In general, this result shows that more accurate clonal information provides better mutation detection.

### Conclusion

We studied the use of clonal information for the purpose of somatic mutation detection and classification in multi-sample whole-genome sequencing data. The proposed statistical framework uses the clusters cellular prevalences and copy number information for detection and classification of low prevalence mutations. Our proposal, MuClone, outperformed other popular mutation detection tools, while providing the added benefit of classifying whole genome sequencing mutations into biologically relevant groups. Both synthetic and real data results showed that using the cellular prevalences of tumour clusters can improve mutation detection sensitivity. Importantly, our results suggest improvement in sensitivity can be achieved without compromising specificity.

Since the accuracy of detecting mutations can affect the performance of phylogenetic analysis, we suggest improvement from using MuClone will impact the field of multi-region sequencing for cancer evolution studies. As the field matures, we expect that the method presented here will be incorporated into more analytically comprehensive modelling of whole genome sequencing data when multiple samples are used to infer properties of clonal dynamics. Next steps are in developing a unified iterative algorithm that alternates between identifying the phylogenetic structure of the constituent clones comprising each tumour sample, and detection of mutations leveraging the new phylogenetic structure.

As sequencing costs continue to decrease (e.g., with Illumina’s NovoSeq platform), multi-sample whole genome sequencing of tumours will continue to proliferate (e.g., rapid autopsy program) as a viable experimental design. Thus, MuClone will be an asset in the arsenal of analytical methods deployed to interpret evolutionary properties of cancer and to gain insights into clonal dynamics in time and space.

## Methods

### Description of MuClone

MuClone uses previously known cellular prevalence information to improve mutation detection and classification. For each sample, MuClone detects mutations from joint analysis of multiple samples. We encode this process in a generative probabilistic framework to perform joint statistical inference of multiple observations (from multiple samples) of the variant allele counts of a mutation of interest. The inputs to the model are: the number of variant reads, and the depth for a set of sequenced loci from multiple samples derived from the same patient; a measure of allele-specific copy number at each locus, in each sample, with tumour content; and the cellular prevalence and the abundance of underlying mutation clusters. MuClone outputs (i) a probability for each locus, at each sample, of being a mutation, and (ii) its cluster.

The probabilistic graphical model of MuClone is depicted in Supplementary Fig. 2.

### Model definition

We first define $g_m^n$ the genotype of a given locus *n* in sample *m*, taking values in ${\cal G} = \{ A,B,AB,AAB,ABB, \ldots \}$. For example, the genotype *ABB* refers to the genotype with one reference allele *A* and two variant allele *B*. For simplicity, we assume the number of reads containing the variant alleles, $b_m^n$ at a given locus with genotype $g_m^n$ and read depth $d_m^n$ follows a Binomial distribution with genotype-specific variant probability $p\left( {g_m^n} \right)$

3 $$b_m^n|d_m^n,p(g_m^n)\sim {\mathrm{Binomial}}\left( {d_m^n,p(g_m^n)} \right).$$

For $g_m^n \in {\cal G}$, the variant probability $p(g_m^n):{\cal G} \to [0,1]$ is defined as

4 $$p(g_m^n) = \left\{ {\begin{array}{*{20}{l}} {\frac{{v(g_{m}^{n})}}{{c(g_{m}^{n})}}} & {v(g_{m}^{n}) \ne 0,v(g_{m}^{n}) \ne c(g_{m}^{n}),} \\ \epsilon & {v(g_{m}^{n}) = 0,} \\ {1 - \epsilon } & {v(g_{m}^{n}) \ne 0,v(g_{m}^{n}) = c(g_{m}^{n}),} \end{array}} \right.$$

where $v(g_m^n):{\cal G} \to {\Bbb N}$ and $c(g_m^n):{\cal G} \to {\Bbb N}$ return the number of the variant allele and the copy number of genotype $g_m^n$ respectively, for example *v*(*ABB*) = 2 and *c*(*ABB*) = 3. The variable $\epsilon \,> \, 0$ is a small positive constant that accounts for sequencing error. It allows for non-zero variant reads, due to sequencing error, when there are no variant alleles in genotype $g_m^n$.

However, since the sequenced reads are independently sampled from an infinite pool of DNA fragments, at a given locus, each read may belong to the normal, reference, or variant population. The normal population stands for normal cells; The reference population are tumour cells which do not have the mutation at the given locus; and the variant population are the ones carrying the mutation. Therefore, using a single genotype state, $g_m^n$, introduces error into our analysis. To account for this fact, we consider using the full genotype state, $\psi _m^n = \left( {{g_N}_m^n,{g_R}_m^n,{g_V}_m^n} \right)$, at a given locus *n* and sample *m*, to model the number of variant reads. Normal population fraction is 1 − *t*_*m*_ where *t*_*m*_ is the tumour content of sample *m* and the cellular prevalence of the mutation is ${\phi} _m^z$ which is the fraction of tumour cells carrying the mutation. According to our prior knowledge, we assume mutations are clustered into *K* clusters. For a given locus *n*, *Z*^*n*^ = *z* ∈ {1, …, *K*} defines which cluster the mutation belongs to. If a position is not a mutation then it belongs to wildtype cluster identified by *Z*^*n*^ = 0.

Therefore, for a mutation at a given locus *n* and sample *m* with cellular prevalence ${\phi} _m^z$ and tumour content *t*_*m*_, the variant allele probability is denoted by $\xi (\psi _m^n,{\phi} _m^z,t_m)$. It is proportional to the sum of the (properly scaled) variant probabilities from each population:

5 $$\begin{array}{*{20}{l}} {\xi ({\psi} _{m}^{n},{\phi} _{m}^{z},{t}_{m})} \hfill & \propto \hfill & {(1 - {t}_{m})c({g_{N}}_{m}^{n})p({g_{N}}_{m}^{n})} \hfill \\ {} \hfill & {} \hfill & { + t_m(1 - {\phi} _{m}^{z})c({g_{R}}_{m}^{n})p({{g}_{R}}_{m}^{n})} \hfill \\ {} \hfill & {} \hfill & { + {t}_{m}{\phi} _{m}^{z}c({g_{V}}_{m}^{n})p({{g}_{V}}_{m}^{n}),} \hfill \end{array}$$

where the first term $(1 - t_m)c({g_N}_m^n)p({g_N}_m^n)$ is proportional to the probability of sampling a read containing variant allele from the normal population, and the second and third terms, $t_m(1 - \phi _m^z)c({g_R}_m^n)p({g_R}_m^n)$ and $t_m\phi _m^zc({g_V}_m^n)p({g_V}_m^n)$, are proportional to the probabilities of sampling a read containing variant alleles from the reference and variant populations, respectively.

Considering the full genotype state, the number of reads containing the variant alleles at a given locus *n* that belongs to cluster *Z*^*n*^ follows a Binomial distribution with probability

6 $$\mu (Z^n) = \left\{ {\begin{array}{*{20}{l}} \epsilon & {{\mathrm{if}}\,Z^n = 0} \\ {\xi (\psi _m^n,\phi _m^z,t_m)} & {{\mathrm{if}}\,Z^n = z\,{\mathrm{and}}\,z \in \{ 1, \ldots ,K\} ,} \end{array}} \right.$$

where $\epsilon$ accounts for sequencing error in wildtype cluster and $\xi (\psi _m^n,\phi _m^z,t_m)$ is the variant alleles probability for *n*th locus, *m*th sample from *z*th cluster. According to Eq. (5), tumour content and cellular prevalence information are incorporated to estimate $\xi (\psi _m^n,\phi _m^z,t_m)$.

Since empirical evidence shows that variant read data is overdispersed, we replace the Binomial model (3) with a BetaBinomial model

7 $$b_m^n|d_m^n,\mu (Z^n),s\sim {\mathrm{BetaBinomial}}\left( {b_{m}^{n}|d_{m}^{n},{\mu} (Z^{n}),s} \right),$$

where *μ*(*Z*^*n*^) is the expected variant alleles probability and the hyperparameter *s* is the precision parameter of the BetaBinomial distribution. The BetaBinomial distribution in Eq. (7) assigns a small chance for mutation when the locus is wildtype, otherwise it is governed by the prior clonal information.

To fully express our model, for each locus, we assume the genotype state follows a categorical distribution with probability vector ${\boldsymbol{\pi }}_m^n \in [0,1]^{|{\cal G}|}$ whose *i*th element is the probability of the *i*th genotype state,

8 $$\psi _m^n|{\boldsymbol{\pi }}_m^n\sim {\mathrm{Categorical}}\left( {{\boldsymbol{\pi }}_m^n} \right).$$

The number of possible genotype states, denoted by $\left| {\cal G} \right|$, is finite given the copy number information. For simplicity, we assume every element of ${\boldsymbol{\pi }}_m^n$ is equal to $\frac{1}{{\left| {\cal G} \right|}}$.

In addition, we also assume that the clonal assignment of a locus, denoted by *Z*^*n*^, follows categorical distribution with probability vector ***τ***:

9 $$Z^n|{\boldsymbol{\tau }}\sim {\mathrm{Categorical}}\left( {\boldsymbol{\tau }} \right).$$

Our probabilistic framework can be succinctly written as

10 $$\begin{array}{rcl}{b_{m}^{n} |d_{m}^{n},\mu (Z^{n}),s} & \sim {{\mathrm{BetaBinomial}}\left( {b_{m}^{n}|d_{m}^{n},\mu (Z^{n}),s} \right),} \\ {{\psi} _m^n|{\boldsymbol{\pi }}_m^n} &\sim {{\mathrm{Categorical}}\left( {{\boldsymbol{\pi }}_{m}^{n}} \right),} \\ {Z^{n}|{\boldsymbol{\tau }}} &\sim {{\mathrm{Categorical}}\left( {\boldsymbol{\tau }} \right)}.\end{array} $$

### Inference

Based on the generative model introduced in (10) mutations are inferred via the posterior probability distribution of a locus *n* belonging to cluster *z*:

11 $$P(Z^{n} = z|b_m^{n},d_{m}^{n},s) \propto {\tau} _{z}\mathop {\prod}\limits_{m = 1}^M {\kern 1pt} \mathop {\sum}\limits_{i \in I} {\kern 1pt} {\pi} _{mi}^n{\cal L}(Z^{n} = z|b_{m}^{n},d_{m}^{n},s),$$

where the variable *i* indexes ${\boldsymbol{\pi }}_m^n$ over the genotype states, ${\boldsymbol{I}} = \left\{ {1 \ldots \left| {\cal G} \right|} \right\}$. The posterior probability of locus *n* belongs to cluster *z* is proportional to the likelihood of observing $b_m^n$ number of nucleotides matching the variant alleles times the prior over tumour cluster *z*. The tumour cluster prior *τ*_*z*_ is the fraction of mutations belonging to cluster *z* and it has been tuned according to wildtype prior; the tumour cluster prior and the cellular prevalence information are encoded in **Ω**. Wildtype prior is our prior information if a locus is a mutation. If we don’t have any information we can set it to 0.5. The likelihood function, ${\cal L}(Z^n = z|b_m^n,d_m^n,s)$, is the BetaBinomial distribution defined in 7.

Based on basic decision theory, a decision can be extracted from a posterior distribution given a loss function. Under the loss function $\ell (z,z\prime ) = {\mathbf{1}}[{\mathbf{1}}[z = 0] \ne {\mathbf{1}}[z\prime = 0]]$, the decision is simply the maximum a posteriori (MAP). That is, if the probability *η* of belonging to any of the tumour clusters is greater than 0.5, we conclude that the locus is mutated in at least one of the *M* samples. The value of *η* is

$$\eta = \mathop {\sum}\limits_{z = 1}^K {\kern 1pt} P(Z^n = z|b_m^n,d_m^n,s).$$

If locus *n* is mutated in at least one of the *M* samples, then the probability of mutation, in each sample, is calculated separately as

12 $$P_{m}^{n}({\mathrm{mutant}}) = \mathop {\sum}\limits_{j \in {\boldsymbol{J}}_{m}^ {\ast} } {\kern 1pt} P(Z^{n} = j|b_{m}^{n},d_{m}^{n},s),$$

where ${\boldsymbol{J}}_m^ \ast$ is the set of clusters of sample *m* whose cellular prevalences are greater than a fixed positive threshold called *Φ*_*T*_,

13 $${\boldsymbol{J}}_m^ {\ast} = \{ j|\phi _{m}^{j} > {\it{\Phi }}_T\} .$$

The threshold *Φ*_*T*_ distinguishes the clusters of sample *m* in which their non-zero cellular prevalence are due to actual variant alleles. The default value of *Φ*_*T*_ is zero. However, depending on the method used for estimating cellular prevalences, it can be set to another positive value, if some non-zero input cellular prevalences indicate wildtype clusters.

In addition, MuClone assigns the locus to cluster z^*^ that maximizes

14 $$z^ {\ast} = \mathop {{{\mathrm{argmax}}}}\limits_{z \in \{ 1, \ldots ,K\} } P(Z^{n} = z|b_{m}^{n},d_{m}^{n},s).$$

This classifies mutations to one of the previously known clusters. The classification of mutations helps in biological interpretation and phylogenetic analysis of the data.

### Code availability

The code is available from https://bitbucket.org/fdorri/muclone.

## Supplementary information

Supplementary Information

## Data Availability

The high grade serous ovarian cancer data used in the current study are available on-line at https://bitbucket.org/fdorri/muclone. All sequencing data has been deposited in the European Genome-Phenome Archive under study accession EGAS00001000547^5^. The NSCLC data used for the analysis is included in suppleantary material of TRACERx study manuscript. Sequencing data is also available for download in the European Genome–Phenome Archive under accession number EGAS00001002247^7^. The patient consent for high grade serous ovarian cancer and NSCLC data are outlined in their primary manuscript^5,7^.
