# Supplementary material for: Somatic mutation detection and classification through probabilistic integration of clonal population information
Source: Commun Biol. 2019 Jan 31;2:44. doi: 10.1038/s42003-019-0291-z (PMC6355807; doi:10.1038/s42003-019-0291-z)
Supplement: Supplementary file 1 — Supplementary Information [file 42003_2019_291_MOESM1_ESM.pdf]

1 Supplementary Figures

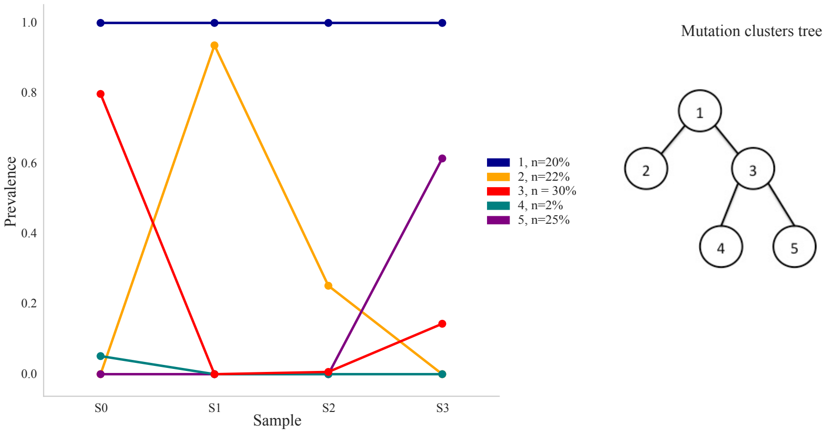

(a) (b)

Supplementary Figure 1: Clonal information example. (a) Cellular prevalences of underlying mutation clusters across multiple samples; (b) The relationship between mutation clusters is represented as a tree

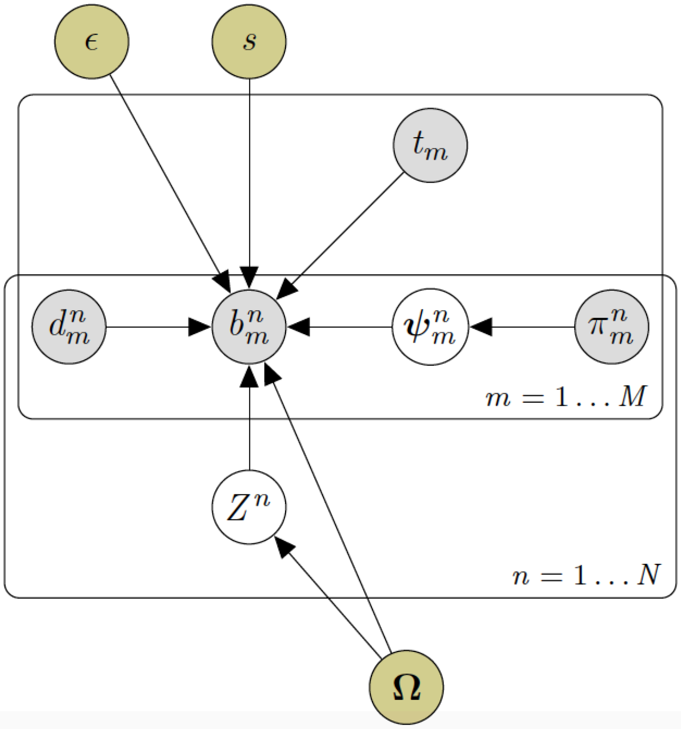

Supplementary Figure 2: Probabilistic graphical model for MuClone: white nodes are unobserved variables; grey shaded nodes are observed variables; golden nodes are external information. The variables $m \in \{1 \ldots M\}$ and $n \in \{1 \ldots N\}$ index the samples and the loci respectively. In sample m, d_m^n is the total number of reads aligned at locus n and b_m^n is the number of aligned reads with B alleles. The genotype state is $\boldsymbol{\psi}_m^n$ and π_m^n is the prior over the genotype states. The tumour content of sample m is t_m and the error rate is ϵ. The parameter s stands for the precision parameter. Tumour clusters prior and their cellular prevalence information are encoded in $\mathbf{\Omega}$ and the variable Z^n denotes the mutation cluster.

2 Supplementary Tables

Patient	Samples	#Validated positions	Anatomic samples
1	6	153	Right Ovary Site 1-4; Omentum Site 1; Small Bowel Site l
2	4	46	Omentum Site 1,2;Right Ovary Site 1,2
3	4	99	Right Ovary Site 1,2;Omentum Site 1; Left Ovary Site 2
4	5	69	Right Ovary Site 1-4;Right Pelvic Side Wall
7	3	59	Left Ovary Site 1; Brain Metastasis; Right Pelvic Mass
9	5	72	Right Ovary Site 1; Left Ovary Site 1; Omentum Site 1,2
10	4	136	Right Ovary Site 1-4

Supplementary Table 1: Summary of high grade serous ovarian cancer data set [1].

Patient	Samples	Cancer type
CRUK0003	4	Adenocarcinoma
CRUK0004	4	Adenocarcinoma
CRUK0005	4	Adenocarcinoma
CRUK0013	5	Adenocarcinoma
CRUK0062	7	Squamous-Cell Carcinoma
CRUK0063	5	Squamous-Cell Carcinoma
CRUK0065	6	Squamous-Cell Carcinoma
CRUK0094	4	Other

Supplementary Table 2: Summary of the NSCLC data set [2].

References

[1] McPherson, A., *et al.*: Divergent modes of clonal spread and intraperitoneal mixing in high-grade serous ovarian cancer. *Nature Genetics* **48**, 758–767 (2016) .

[2] Jamal-Hanjani, M., *et al.*: Tracking the evolution of non–small-cell lung cancer. *New England Journal of Medicine* **367**(22), 2109–2121 (2017) .
